# Implementing the My Positive Health dialogue tool for children with a chronic condition: barriers and facilitators

**DOI:** 10.1186/s12887-024-05258-0

**Published:** 2025-03-05

**Authors:** S. de Jong-Witjes, E. E. Berkelbach van der Sprenkel, M. C. Kars, M. Huber, S. L. Nijhof, R. Nuboer, D. M. Broekhuijsen-van Henten, C. A. Lasham, E. G.A.H. van Mil, E. M. van de Putte

**Affiliations:** 1 Department of Primary and Community Care, Radboud University Medical Centre, Nijmegen, the Netherlands; 2 Department of Pediatrics, Wilhelmina Children’s Hospital, University Medical Center Utrecht, Utrecht, The Netherlands; 3 Julius Centre for Health Sciences and Primary Care, University Medical Centre Utrecht, Utrecht, The Netherlands; 4 Institute for Positive Health, Utrecht, The Netherlands; 5 Department of Pediatrics, Meander Medisch Centrum, Amersfoort, The Netherlands; 6 Department of Pediatrics, Isala, Zwolle, The Netherlands; 7 Department of Pediatrics, Tergooi Medisch Centrum, Hilversum, The Netherlands; 8 Department of Pediatrics, Jeroen Bosch Ziekenhuis, ‘s-Hertogenbosch, The Netherlands

**Keywords:** Positive Health, Pediatrics, Shared decision making, Pediatric chronic conditions, Person-centered care, Child participation

## Abstract

**Background:**

The My Positive Health (MPH) dialogue tool for children was developed to aid children and teenagers in reflecting and communicating about their health from a broader perspective. This study investigates facilitators and barriers to implementation in pediatric care and assesses experiences of healthcare professionals (HCPs) and children regarding effectiveness.

**Methods:**

We conducted a mixed-methods study involving six Dutch pediatric outpatient clinics. Quantitative data on facilitators and barriers were obtained from 18 out of 20 participating HCPs (pediatricians, nurse practitioners and physician assistants) using the Measurement Instrument for Determinants of Innovations. Additionally, qualitative insights were gathered through semi-structured interviews with 17 HCPs and 30 children (8–18 years old) with chronic conditions.

**Results:**

Facilitators identified in both user and innovation domains included improved patient understanding and the tool’s simplicity, while barriers involved organizational constraints and integration issues, for example limited resources and lack of organizational support. Participating HCPs highlighted the tool’s role in fostering person-centered conversations, especially for children with chronic conditions. Children positively viewed the tool, noting its ability to enable deeper, personalized interactions with HCPs.

**Conclusion:**

This study on the implementation of the MPH dialogue tool for children in pediatric care highlights its user-friendliness and relevance, alongside challenges like organizational constraints. Beneficial for person-centered care and children’s active participation, the tool enhanced healthcare dialogues and empowered children in their health journey. However, HCPs faced integration challenges within existing practices. Addressing these barriers and providing organizational support are vital for effectively implementing the MPH dialogue tool and optimizing pediatric patient engagement and care quality.

**Supplementary Information:**

The online version contains supplementary material available at 10.1186/s12887-024-05258-0.

## Introduction

In 2017, the My Positive Health (MPH) dialogue tool for children was developed following a qualitative study involving 65 children and teenagers, with and without chronic conditions, to explore their perception of health [1–3]. This tool aims to help them to gain insight into their health priorities and express aspects they wish to change, to ultimately enhance their participation during consultations with healthcare professionals (HCPs). In this manuscript, the term ‘children’ is used to encompass both children and teenagers between 8 and 18 years.

Pediatric patient engagement – defined as the active involvement of patients in their own healthcare process - is increasingly receiving attention, with children valuing active communication and information-sharing with HCPs to gain more control over their healthcare experiences [4–6]. In addition, they prefer to be involved in decisions regarding their treatment, and even young patients may have a personal agenda and a wish to participate during visits to their HCP by sharing their subjective experiences with health and illness [5, 7, 8]. Social engagement through small talk and considering the child’s perspective before addressing medical topic(s) can boost participation, foster self-confidence, and aid in developing a sense of autonomy and control [9, 10]. In the long term, pediatric patient engagement can lead to improved health outcomes, better treatment adherence, enhanced health literacy, and more effective self-management skills [4, 5, 7, 9].

Despite this, child participation in medical consultations remains limited and there has been little improvement over the past 50 years [11]. Few innovations exist to promote child participation during clinical encounters, highlighting the need for interventions enabling children to express their thoughts and opinions [11–13]. Integrating innovations in clinical settings is a complex challenge, often complicated by the oversight of crucial implementation data, including both barriers and facilitators. This oversight leads to suboptimal clinical benefits for HCPs, patients, and other stakeholders [14, 15].

To design an evidence-based implementation strategy informed by context and stakeholder input, this study aimed to identify barriers and facilitators to implementing the MPH dialogue tool in pediatric healthcare. Our secondary objective was to assess the tool’s effectiveness, as perceived by both HCPs and children, during implementation of the of the MPH dialogue tool within six Dutch hospitals’ pediatric care departments.

## Methods

We conducted this study to assess the MPH dialogue tool for children and identify implementation barriers and facilitators in six Dutch hospital pediatric outpatient clinics (one academic and five non-academic). We followed the Standards for Reporting Qualitative Research (SRQR) and Standards for Reporting Implementation Studies (STARI) to enhance the reporting process [16, 17].

### Study design

Typically, implementation research designs are ‘hybrid’ in nature, combining elements of implementation and effectiveness research to promote the integration of research findings into routine practice [14, 15]. For this study, a type three hybrid design was applied which mainly focusses on implementation outcomes while also collecting effectiveness outcomes that relate to uptake of the intervention [15]. We applied a combination of informational, motivational, educational, and organizational strategies adopted from the Netherlands Organization for Health Research and Development (ZonMw) implementation plan [18]. To assess the implementation strategy and intervention effectiveness, we employed a mixed-method approach including questionnaires and semi-structured interviews.

### Innovation

**Fig. 1:**
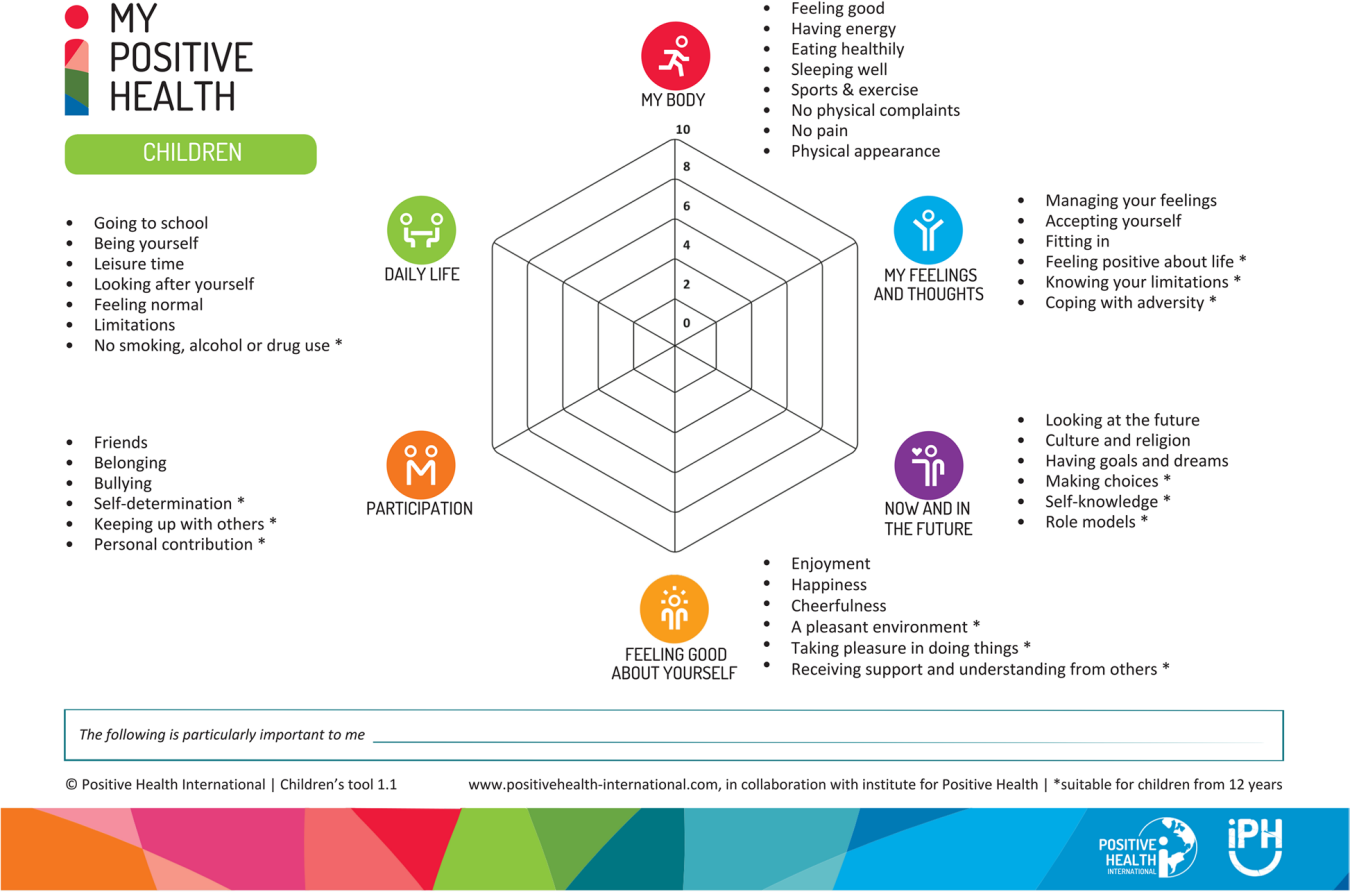
Dimensions and themes of the MPH dialogue tool for children

The MPH dialogue tool for children comprises six dimensions displayed in a spider web chart, encompassing 39 aspects related to health (Fig. 1). It is designed to guide children between 8–18 years old in reflecting on their health, and is available in both paper and digital formats (accessible online at https://positivehealth-international.com/dialogue-tools/). The MPH dialogue tool offers an overview of a child’s self-reported health status and can be employed, for instance, during dialogues with HCPs to support in providing appropriate care. Participants were asked to complete the spider web chart, rating their satisfaction across six dimensions of health. These ratings served as the foundation for in-depth, person-centered discussions that explored areas of strength or concern identified by the children. The conversations focused on setting goals and strategies that aligned with the child’s values and priorities. Details on the development of the dialogue tool can be found in the article by de Jong-Witjes et al. [3]. Participating HCPs underwent a training that consisted of two interactive online sessions, with each session lasting 2 hours, to ensure correct use of the tool. They attended a third follow-up meeting focusing on further implementation and practical application. During these sessions, HCPs were introduced to the concept of ‘Positive Health’ and had the opportunity to practice using the MPH dialogue tool themselves. Through peer-to-peer coaching, they developed skills for conducting “alternative dialogues” which emphasized active listening, coaching, and the use of open-ended questions [19]. The aim was to empower children to identify their own solutions, with HCPs facilitating rather than directing the conversation, ultimately leading to active steps and follow-up as necessary.

### Participants

A convenience sample of pediatric HCPs were recruited via email from the Wilhelmina Children’s Hospital, part of the University Medical Center in Utrecht, and from five regional general hospitals. Criterion based sampling was used to recruit patients that were invited by their own HCP if they met the inclusion criteria (Table 1). If the child and/or their parent(s) gave permission, they were contacted by the researcher to inform them about the study. Both children and their parents provided written assent and/or informed consent upon participation in accordance with Dutch regulations [20]. The Medical Research Ethics Committee of the University Medical Centre Utrecht classified this study as exempt according to the Medical Research Involving Human Subjects Act (MREC protocol number 20–790/C).

**Table 1:** Inclusion criteria

Inclusion criteria
Age: 8–18 years
Chronic condition, currently in stable condition^a^
Scheduled for regular appointment with pediatric HCP
Able to read and fill out the dialogue tool’s corresponding questionnaire in Dutch

^a^This was defined as a disease that has already been diagnosed and does not cause any acute threat to the child at the moment

### Data collection

Data collection was divided into two implementation cycles that took place from January to August 2021. The first cycle, from January to April 2021, involved HCPs from two hospitals. The second cycle, from May to August 2021, included HCPs from four additional hospitals. Each cycle began with training the HCPs, followed by patient recruitment and integration of the MPH dialogue tool into consultations. The researcher provided digital dialogue tool instructions to children and parents after HCP invitation. The MPH dialogue tool could be completed either on paper or digitally via the Positive Health website and printed prior to the consultation. No personal data were stored. HCPs were interviewed virtually at the end of the three-month cycle, followed by a digital invitation to complete an online questionnaire. The virtual interview with the child occurred shortly after the consultation, typically within a week, to avoid recall bias.

#### Quantitative data collection

Understanding the various determinants that influence the process and outcome of implementation is essential for devising tailored strategies that ensure successful implementation [21]. We utilized the Measurement Instrument for Determinants of Innovations (MIDI), a tool designed for implementation research. The MIDI gathers insights into how specific factors may impact desired practice changes by focusing on the HCP perspective to pinpoint innovation barriers and facilitators. The MIDI comprises four domains: innovation (7 items), the user (11 items), the organization (10 items), and the socio-political context (1 item). All 29 questions have a response scale ranging from 1 (totally disagree) to 5 (totally agree). We slightly modified the validated questionnaire in accordance with the MIDI-regulations, for our study’s context [21]. As this study was limited to a single country, the social-political context was not expected to differentiate and thus removed. Additionally, since this was a pilot study we omitted items 14, 15, and 20 from the questionnaire. These items respectively addressed ‘descriptive norm’ (the extent to which colleagues use the innovation), ‘subjective norm’ (the influence of important others on the individual’s use of the innovation), and ‘replacement of staff leaving the organization’. Castor Electronic Data Capture software was used to collect this data. The reliability of the MIDI questionnaire was good (α = 0.81).

#### Qualitative data collection

Semi-structured interviews were conducted with both HCPs and children, beginning with an open-ended question: ‘*What was your experience with using the MPH dialogue tool for children during the consultation?*’. Hereafter, we used the following interview guides that were largely based on the determinants of the MIDI (Appendix A). This approach ensured comprehensive coverage of all factors influencing the implementation, in addition, it offered participants an opportunity to elaborate on specific determinants and allowed for in-depth exploration of barriers and facilitators to implementation.

The interview guide for children was extended with several general open-ended questions to assess the effectiveness of the MPH dialogue tool in practice. These additional questions, drafted by the research team, aimed to explore the tool’s impact on three key areas: experiencing directorship, awareness of health as a broad concept, and the person-centered consultation. In terms of experiencing directorship - defined as the process of making intentional choices, setting goals aligned with personal values and desires, and taking ownership of one’s actions and their outcomes - questions assessed whether the MPH dialogue tool influenced children’s preparation and priorities for their medical consultations. Regarding awareness, inquiries focused on how children perceive their health, in general and in the context of using the tool. Finally, we asked whether children felt their views were acknowledged and understood, exploring the person-centeredness of the consultation and the perceived quality of communication with doctors and nurses. Children shared their experience based on memory, but the researcher had a blank paper version of the tool at hand to help the children recall their experience if needed.

Interviews were conducted via Microsoft Teams video conferencing due to social restrictions during the COVID-19 pandemic. Children generally answered questions independently, with the option for parents to be present or participate if preferred. Before starting the interview, informed consent was reaffirmed, ensuring that the participant fully understood the study and its procedures. Interviews were recorded and transcribed verbatim, and personal information was anonymized during transcription. The data was securely stored on encrypted servers with restricted access, available only to the research team in line with institutional data protection protocols.

### Data analysis

Data analysis was performed by one researcher (SdJW) under supervision of the research team that consisted of a research pediatrician as principal investigator (EvdP), a senior qualitative researcher (MCK), and the founder of the Positive Health philosophy (MH). This research team was consulted at least every six weeks to discuss study status and recent findings.

#### Quantitative analysis

Quantitative data from the MIDI-questionnaires were analyzed using SPSS 26.0. Descriptive statistics, including mean, standard deviation, range, and percentage, were used to evaluate HCP-reported barriers and facilitators for implementing the MPH dialogue tool. Consistent with prior studies, responses of ‘totally disagree/disagree’ from ≥ 20% of participants indicated barriers, while ‘agree/totally agree’ from ≥ 80% signified facilitators [22]. Cronbach’s alpha was calculated to assess the questionnaire’s internal consistency.

#### Qualitative analysis

The interviews with children and HCPs were analyzed individually and at various time points. A generic approach using interpretive description was employed [23]. The process began with the researcher (SdJW) and a junior researcher (JS or FW) independently reading the transcripts. A preliminary code tree was created by the research team, including a senior qualitative researcher (MCK), based on an inductive coding process and a deductive template of codes derived from the MIDI. This code tree was refined during bi-weekly reviews where new codes were integrated and discrepancies were resolved. These bi-weekly reviews facilitated a comparative and iterative process, with insights and findings continuously informing subsequent rounds of data collection and analysis. Coding saturation was reached when no new themes could be identified. MAXQDA 2020 supported this coding process as detailed in appendix B. After analyzing the first implementation cycle, the researcher summarized key findings illustrated with representative quotes, which were then validated by the entire research team. Based on these results, the research team provided insights and recommendations for the second implementation cycle.

## Results

### Participants

In this study, 20 HCPs participated (Table 2), including 18 who completed the MIDI questionnaire and 17 who were interviewed. These HCPs consisted of doctors, nurses, and physician assistants (Table 2). Of the 30 children, 18 participated during the first implementation cycle and 12 during the second implementation cycle. Table 3 shows the characteristics of the participating children.

**Table 2:** Characteristics of participating HCPs

	*N*	%
Total	20	100
*Gender*		
Female	16	80
Male	4	20
*Profession*		
Pediatrician	10	50
Nurse practitioner	6	30
Physician Assistant	4	20
*Subspecialty*		
General pediatrics	6	30
Pulmonary diseases & allergology	4	20
Endocrinology	4	20
Gastroenterology	3	15
Social pediatrics	2	10
Nephrology	1	5

**Table 3:** Characteristics of the participating children

	*N*	%
Total	30	100
*Gender*		
Female	15	50
Male	15	50
*Age in years*		
8–11	7	23.3
12–18	23	76.7
*Diagnosis*		
Respiratory diseases	7	23.3
Diabetes	6	20
Gastro-intestinal diseases	6	20
Juvenile Rheumatism	3	10
Renal diseases	2	6.7
Allergies	2	6.7
Eye conditions	1	3.3
Chronic fatigue	1	3.3
Benign tumor	1	3.3
Medically Unexplained Physical Symptoms	1	3.3
*Time in care (since diagnosis)*		
0–6 months	2	6.7
6–12 months	1	3.3
1–3 years	12	40.0
4–5 years	5	16.7
>5 years	10	33.3
*Healthcare professional involved in consultation*		
Pediatrician	15	50
Physician assistant	6	20
Nurse practitioner	9	30

### Barriers and facilitators to implementation

#### Quantitative results - HCPs

Seven barriers and six facilitators were identified based on the analysis (Table 4). The facilitators were identified within the user domain (four facilitators) and the innovation domain (two facilitators). The barriers were identified within the innovation domain (two barriers) and the organization domain (five barriers). Appendix C provides detailed insights into the determinants assessed and the corresponding questions.

**Table 4:** Barriers and facilitators

	Domain	(Totally) disagree (%)	(Totally) agree (%)
**Facilitators**			
4. Complexity^a^ *The MPH dialogue tool for children is too complex for me to use.*	Innovation	100	
7. Relevance for patient *I think the MPH dialogue tool for children is relevant for my patients.*	Innovation		83
8. Personal benefits/drawbacks *- Using the MPH dialogue tool for children helps me better understand what is important to my patients regarding their health.*	User		89
9. Outcome expectations *I expect that using the MPH dialogue tool for children will actually achieve the following objective for my patient:* -The patient develops a broader view of health.	User		94
13. Social support *I can count on adequate assistance from my colleagues if I need it to use the MPH dialogue tool for children.*	User		83
17. Knowledge *I have sufficient knowledge to be able to use the MPH dialogue tool for children.*	User		83
**Barriers**			
3. Completeness *The MPH dialogue tool for children provides all the information and materials needed to work with it properly.*	Innovation	28	
5. Compatibility *The MPH dialogue tool for children fits well with how I am used to working.*	Innovation	22	
23. Time available *The time available is sufficient to integrate the MPH dialogue tool for children into my daily work as intended.*	Organization	33	
24. Material resources and facilities *Our organization provides me with enough materials and other resources or facilities to use the MPH dialogue tool for children as intended.*	Organization	22	
25. Coordinator *In my organization*,* one or more persons have been designated to coordinate the process of implementation of the MPH dialogue tool for children.*	Organization	28	
27. Information accessible about use of the innovation *I have easy access in my organization to information on the use of the MPH dialogue tool for children.*	Organization	22	
28. Performance feedback *In my organization*,* there is regular feedback on the progress of the implementation of the MPH dialogue tool for children.*	Organization	61	

^a^In most cases, determinants showed positive associations with usage – higher scores indicating greater expected usage. However, when this pattern did not apply, determinants were marked with an asterisk and inversely scored

#### Qualitative results - HCPs

In this section, we will clarify and illustrate the identified barriers and facilitators using interview content, while focusing on the levels of the innovation, the user, and the organization.

##### Innovation

General experiences

Most HCPs reported positive experiences stating that the tool often prompted a different kind of conversation (Table 5, Q1). HCPs also noted that it assisted in developing a deeper understanding of their patients on a more personal level and in better addressing their patients’ needs (Q2).

**Table 5:** Quotes from the healthcare professionals

Domain	Topic	Quotes
**Innovation**	**General experience(s)**	**Q1** ‘And that really brings many surprises…. almost every time something totally different has come up, different than I expected. So really, I found it … I think I’m always focused on more than just the disease anyway, but the child always gives a socially desirable answer. “How’s school going?” “Good.” and that’s it. And if I don’t go further into it then. [.] and this is really much more about: what’s important?’ *Pediatrician*
		**Q2** ‘Well very nice, very insightful, very nice conversations, it’s just… the big difference is that it’s actually not about medical things, which you normally talk about, but just about everyday things. So the distance between doctor and patient does really become smaller because of that, I think.’ *Pediatrician*
	**Completeness**	**Q3** ‘… because of course we practiced that [during the training]. But each time, I was searching for where the questions [to guide or structure the conversation] were again, because often I couldn’t find them.’ *Pediatrician*
		**Q4** ‘I did see that the digital version contained more questions. And that is easier for the children to fill in, I think, and that is less the case with only the paper [with the spider web chart]. So that… so I think that the digital version gives a better, more honest representation of the situation. Because the questions [aspects] are worked out there.’ *Nurse practitioner*
	**Compatibility**	**Q5** ‘But … and I think that we all experienced how hard it is to stay silent. To say nothing, to wait for the other person…. You want to help so much and fill in.’ *Physician Assistant*
		**Q6** ‘Well and then the question is: when you know, where… where do you leave it? Where do you store it? What makes you able to talk about it again next time? […] You actually want something linked to the electronic patient file or something so you can access it easily.’ *Physician Assistant*
		**Q7** ‘We just have too many patients scheduled each afternoon to always have this kind of conversation, so then you really have to, which I did now, just have patients come back … or I just called them, to do that conversation.’ *Pediatrician*
		**Q8** ‘… so what I’m still trying to figure out a little bit is, is say you have the patients’ agenda based on the dialogue tool and you also have your agenda with the medical things that you want to discuss. How do you weave that together? Yeah… I haven’t quite figured that out yet. […] I still think. because you don’t want them to be two separate things, do you? You actually just want to integrate them.’ *Pediatrician*
	**Relevance**	**Q9** ‘Yes, I noticed the children liked it too from how they, how much they enjoyed filling in things themselves and talking about it. […] especially with teenagers, I think. Because you want them to. to become more aware of what, what. that they become more engaged with their lives themselves and realize that this illness is theirs and not their parents.’ *Nurse practitioner*
		**Q10** ‘And certainly with children you have been treating for a long time, so you know quite a bit about them, but certainly also the more… quiet children, or the children from socially disadvantaged backgrounds who perhaps don’t always speak their mind freely, it’s really nice to look at them in a different way for once. And to look more at what they actually want and actually find important.’ *Pediatrician*
	**Complexity**	**Q11** ‘Yes, I think so, the spider web chart in itself was clear […] I didn’t find it difficult to start a conversation with it. [.] The younger children indicated that that was quite difficult for them, whereas the teenagers, well they often indicated that it helped them to think about it beforehand.’ *Physician Assistant*
		**Q12** ‘Yes, so in itself, it’s quite. I find it quite an easy tool […] And then you can easily have a conversation about that as well.’ *Pediatrician*
		**Q13** ‘So yeah, he thought everything was going well and that all was good, and he had high scores everywhere [all dimensions]. And it did feel to him as kind of a report card of sorts, thinking he shouldn’t score low anywhere, you know. Because that would mean he didn’t do well […] So I thought that was kind of a tricky one.’ *Physician Assistant*
**User**	**Personal benefits**	**Q14** ‘But it, it does sometimes really add something … I think particularly the bond that you have with a patient and that at a certain point you start to delve a little deeper to see what else is going on. And then maybe it’s not always necessarily contributing content in terms of the disease you’re treating, but in terms of the patient as a person. *Pediatrician*
		**Q15** ‘And I think that it lends itself extremely well to help me to see more of the child rather than just the disease. So we always do try to ask about some background of a child and inform: what interests does the child have? Who does his family consist of? How is he doing at school? And I think that precisely because of the MPH dialogue tool you get different conversations, more focused on the child itself.’ *Physician Assistant*
		**Q16** ‘And also gives some insights. I also had a mother saying: ‘’We never really talk about this at home. Well funny that you talk about it like that, about friends or being alone.’’ Yes, it was really nice. But anyway, again, it’s very nice. On the other hand I think: yes, well. the GP could also do this, so to speak.’ *Pediatrician*
		**Q17** ‘And yes, especially if there are things that don’t really have anything to do with their disease or anything medical, then at most you can think about it with them or trigger them to actually solve the issue themselves, because that’s also intent, of course: what do you want more of and how are you going to do that? But then again, am I, as a Pediatrician the person to do that? On the other hand, if I am not, who is? So there … that’s one of the questions, that I also struggle with a little bit.’ *Pediatrician*
		**Q18** ‘I think that we as pediatric nurses are actually a bit, well. also a ‘spider in the web’, so to speak. We should be able to act in all areas a bit. But the moment it becomes something very complicated in the psychosocial area, for example, then it is our job to refer. […] So in that sense I think that…well, at least I think that is part of our task to gather this information.’ *Nurse practitioner*
	**Outcome expectations**	**Q19** ‘Right, especially with teenagers, I think. […]I think that the MPH dialogue tool contributes very nicely to this, that they themselves think about it more. […] Because they determine, what is important to them and how are they going to… How do they want to do that and how? What do they need for that? And yes that’s all part of self-management of course, that they think about it themselves without us immediately starting to give answers to that.’ *Nurse practitioner*
		**Q20** ‘I think that if you let them choose the topic of conversation each time, what they find important in the conversation, then at a certain point they might dare to come up with more questions regarding their treatment. Now it is often the case that the doctor asks the questions and the patient answers. And if you systematically turn that around, I think you get a completely different culture, and so there is more room for questions.’ *Physician Assistant*
	**Social support**	**Q21** ‘In particular, we did talk a few times about… she [colleague] talked about a very nice conversation she had had, in which she was very proud that she had remained very quiet and in which something very nice had indeed come out of it, in the end. So she was really kind of proud of how that went. So then of course it’s nice to share those experiences.’ *Pediatrician*
		**Q22** ‘Um, yes, I do think that it takes time, but I do think that it can work. I am optimistic about that. That also has to do with the fact that the other two colleagues who are participating in the study are both very driven.[…] And I don’t think ultimately everybody’s going to use it. You know, I think there will also be colleagues who aren’t going to use it. […] And I have to practice more myself first to just get more dexterity, more experience in it, because then I can, I think, with the examples I think I can ‘win people over’ as well.’ *Pediatrician*
	**Knowledge**	**Q23** ‘Also the training is of course organized in a special way in this era, via videocall and you name it. But I think still it was well organized and I did learn a lot from that. I did think it was well set up and I gained both skill and as well as knowledge yes.’ *Physician Assistant*
		**Q24** ‘I think the training is a very nice start, and very interactive. But the interview technique, so to speak, requires a bit more for me… because that well… to practice it properly or to have sample questions I can ask. […] It does take some time to understand, also from the child’s perspective: knowing what was asked of the child and what the child had to had to fill in. ' *Physician Assistant*
**Organization**	**Time**	**Q25** ‘If you make it, I think, part of your way of working, I don’t think so. Because then you end up teaching those children to bring to the table their things that they believe are important, and so you will always get everything covered. In the beginning I think it does take more time, because then it requires more investment from yourself. But I don’t think necessarily your consultation hours have to lengthen very much once you get the hang of this.’ *Physician Assistant*
	**Organizational support**	**Q26** ‘Because we have to, I think … what we have to do now is just kind of see how we can implement that in our organization and show ourselves that it’s going well. Then we can get our colleagues excited about it.’ *Pediatrician*

Completeness

Despite training in communication skills, some HCPs desired additional guidance on conversation structure, and they expressed a need for a brief instruction or manual (Q3). They also preferred direct access to children’s individual responses on the 39-item questionnaire over just the summary spider web chart for guiding discussions (Q4).

Compatibility

In the HCP training, emphasis was placed on valuing and allowing for pauses of silence, recognizing that children require time to formulate their responses, and initiate interaction. The HCPs revisited this topic during the interviews by noting the challenge of adapting to this approach as it differed from their usual practice (Q5). However, most HCPs were confident in their ability to adapt to this new approach, not perceiving it as an obstacle in using the dialogue tool. Questions arose regarding the integration of the tool into the electronic patient file, highlighting its potential for streamlined use and enhanced documentation in medical records (Q6). Related to compatibility, the primary concern was the significant time investment needed, especially in the initial stages, to integrate the new tool into the consultation process (Q7). Nevertheless, most HCPs were optimistic that with time and increasing familiarity with the dialogue tool, they would develop effective methods to incorporate it into their work process. Another issue highlighted in the interviews was the potential conflict between using the tool and adhering to the (predetermined) consultation agenda of HCPs. Some HCPs expressed difficulty in aligning their standard agenda with the children’s needs as directed by the dialogue tool (Q8). One HCP solved this by thoroughly preparing and focusing on just one or two key aspects of the tool (those most important to the child) per session.

Relevance

More than 80% of participating HCPs agreed that the MPH dialogue tool is relevant for their patients, identifying ‘relevance’ as a key facilitator (Q9-10). The HCPs explained this relevance varies depending on patient characteristics, such as age, condition, and ability or willingness to engage in consultation. HCPs emphasized the tool’s greater utility for children with chronic conditions and during follow-up appointments rather than in an acute setting.

Complexity

Most HCPs, viewing complexity from their own perspective, found the tool user-friendly, and they particularly appreciated the spider web chart’s visual ease of use (Q11-12). However, when considering the children’s viewpoint, some HCPs observed challenges for eight- to twelve-year-olds in completing the questionnaire online. Moreover, according to the HCP’s, these younger children had difficulty understanding the purpose of the tool as a conversation facilitator about health, rather than a health rating system (Q11-13). Because of this, HCPs noted the risk of the tool being misconstrued as a means to measure and achieve optimal health status.

##### User

Personal benefit(s)

Regarding the user, key facilitators identified were the personal benefits derived from using the tool. HCPs frequently cited that gaining new insights and a richer, more comprehensive understanding of their patients was a major personal benefit (Q14-15). In addition, the majority of the HCPs recognized the deepening of conversations and the resultant stronger personal bond with their patients as another significant benefit. In contrast, one HCP questioned the practical utility of these newfound insights and wondered if they might be more relevant to a general practitioner (Q16). This relates to the determinant of ‘professional obligation’ and the alignment of the innovation with the responsibilities HCPs perceive in their roles. Several HCPs expressed doubts about discussing all health-related topics covered by the dialogue tool as they considered it their primary responsibility to focus on the specific condition for which the child was receiving medical care (Q17). On the other hand, some HCPs considered identifying other health-related problems as part of their professional task and saw potential value in the dialogue tool for this purpose (Q18).

Outcome expectations

During interviews, HCPs evaluated the likelihood and significance of achieving the goals as intended by the innovation. They discussed objectives such as enhancing (health) awareness, gaining insight or inducing self-reflection (Q19). A few HCPs commented that use of the dialogue tool should not be expected to directly influence patient health, and instead suggested it should be considered a supplement to standard care for promoting health awareness. Several HCPs mentioned the tools’ potential to broaden health perspectives. One of the elements that attributes to this is the tools’ emphasis on the connections between different health dimensions. Particularly for teenagers, HCPs saw the tool as beneficial in helping them realize what influence they (can) have on their own health. Many HCP’s recognized a noticeable shift towards prioritizing the child’s agenda during consultations, and they viewed this as a form of empowerment. On the other hand, some HCPs were critical of outcomes such as self-management and empowerment, contemplating that consistent and repeated use of the dialogue tool would be essential to genuinely nurture these aspects. (Q20). Additionally, challenges were noted regarding the role of parents, especially when they tended to dominate the conversation.

Social support

Social support refers to the assistance that users receive or anticipate from significant social contacts, such as colleagues, fellow professionals, department heads or management, during innovation adoption. Most HCPs received beneficial support from colleagues, particularly those participating in the implementation study, noting that being able to deliberate and share experiences boosted their enthusiasm and motivation for the dialogue tool (Q21). Most HCPs felt that colleagues not yet using the tool would embrace it after gaining experience with it and seeing tangible implementation results (Q22).

Knowledge

Quantitative data revealed that over 80% of HCPs felt knowledgeable enough to use the MPH dialogue tool for children, and most of them agreed that the training provided them with the necessary knowledge and a solid foundation for its use (Q23). Interviews confirmed that nearly all HCPs were well-informed about the tool’s content, and one HCP stressed the importance of knowing all aspects of the tool to fully comprehend what was asked of the children and to adjust their communication techniques accordingly (Q24).

##### Organization

Time and Organizational Support

Time and financial resources were central themes when talking about organization-related determinants. HCPs noted that using the dialogue tool often extended consultation times due to discussions on new health topics, which would sometimes lead to longer sessions, referrals, or treatment plan adjustments. However, one HCP observed that the tool could save time by addressing the child’s concerns early in the consultation, avoiding last-minute questions. As previously stated, some HCPs mentioned that while the tool initially added time to consultations, they did not expect this to cause prolonged sessions in the long term because both children and HCPs would become accustomed to this format and the alternative dialogue (Q25). The MIDI questionnaire identified additional organizational barriers, such as material resources, facilities, coordination, information accessibility, and performance feedback. These were framed in relation to HCP’s own organizations (e.g., ‘*Our organization provides me with sufficient materials and facilities to use the MPH dialogue tool as intended’*). In this implementation study, which was coordinated by a single academic medical center, HCPs in regional general hospitals reported that relevant information, materials, and facilities were less readily available. They highlighted the necessity of adequate organizational support to effectively integrate the dialogue tool into consultations (Q26).

### Effectiveness

#### Qualitative results – children

In this section, we will clarify and illustrate the children’s experience regarding the three key areas: experiencing directorship, awareness of health as a broad concept, and the person-centered consultation.

##### Experiencing directorship

Most children reported that they felt comfortable discussing various health aspects using the dialogue tool. They reported feeling the autonomy to choose which topics to address. Some mentioned it enabled them to guide the conversation or noticed that they were asked what they wished to discuss (Table 6, Q1-2). Although a majority of the interviewed children did not plan specific topics for discussion with their HCP, many seemed to have reflected on their own health prior to the consultation. On the other hand, a few children had specifically considered their discussion topics in advance and found the dialogue tool helpful in this regard (Q3-4). The tool provided structure as its visual elements, like the spider web chart, made different dimensions and aspects clear. Additionally, children spontaneously mentioned two topics without introduction by the researcher: goal setting and desire for change. Some mentioned their conversations had led to formulating specific health goals, often related to making health-related adjustments. The visual aspect was particularly noted as helpful in this regard. In terms of desire for change, children used the tool to identify areas for improvement to discuss with their caregivers (Q5-6).

**Table 6:** Quotes from the children

Domain	Topic	Quotes
**Directorship**	**Setting the agenda**	**Q1** ‘Yes … and I think if you apply it, you don’t necessarily have to talk about it, but you can if you want.’ *Boy with asthma*,* 17 years old.*
		**Q2** ‘She [HCP] said: what do you want to talk about? And then I said: about “the future”, because I really didn’t know what it would all be like later.[…] I liked that, because normally it’s like: how is it going with this and how is it going with that? And now it was more of: I do want to talk about this and that, instead of these other things. […] Yes, I do think that it helped me a lot and I found it more pleasant now, because it was difficult for me to explain my worries about the future… and we had never really talked about that before.’ *Boy with asthma*,* 12 years old.*
	**Preparation**	**Q3** ‘Well I actually thought: I’ll see what [name of HCP] wants to know and then I’ll just answer that. I didn’t really have anything I wanted to discuss myself actually.’ *Boy with inflammatory bowel disease*,* 15 years old.*
		**Q4** ‘Because you are a little better prepared for what you want to say or want to ask anyway.’ *Girl with chronic fatigue*,* 15 years old.*
	**Goalsetting and change**	**Q5** ‘Well, to also see for myself if it ends up getting better with certain parts [aspects] or worse, to work on that more, I guess.’ *Girl with juvenile rheumatoid arthritis*,* 18 years old.*
		**Q6** ‘It was kind of useful, because I actually get more insight myself, because I never really thought about… about how I feel and so on… and so… you can actually just talk a little bit and then see if that’s something you want improve or something you can do better and so on. so to speak.’ *Boy with Crohn’s disease*,* 17 years old.*
**Awareness**	**Reflection**	**Q7** ‘Well there were a few questions of which I thought: well, I’ve never actually thought about that in that way, but then because of this you think about it a little bit more. […] Well, I liked it, because this way you look at your health differently and you realize how healthy you are.’ *Boy with asthma*,* 14 years old.*
		**Q8** ‘She [HCP] asked why the score was lower. Then I started thinking: yes, why is it lower? Yes, I did get a realization: that is because of that or because of that. […] So still just kind of the clarity you get that you really see it in a diagram. Yeah, I really started thinking about it like: yeah, what why does the spider web chart look like that?’ *Girl with diabetes*,* 17 years old.*
	**Outcome expectations**	**Q9** ‘For me the web was kind of big, so high points. But yes, I can see from my own experience that I’m doing just fine. I know that too, but it’s a confirmation that everything is going well with me.’ *Girl with Crohn’s disease*,* 16 years old.*
		**Q10** ‘It’s also kind of nice for yourself to kind of see where you stand and then sometimes you see things that you didn’t expect… at least things that come out that you didn’t expect.’ *Girl with allergies*,* 17 years old.*
**Consultation**	**Experience**	**Q11** ‘I did find it difficult, yes, because I was like … yes, I did give those answers myself [in the questionnaire] but I still found it difficult to think of why did I fill that out.’ *Girl with medically unexplained physical symptoms*,* 17 years old.*
		**Q12** ‘For me it was not very different…um it was just a little more…there were a little more open-ended questions. So we just went a little deeper into it, so to speak, because I don’t know… usually the conversations are about the same thing and I don’t really get those questions and now it was also about other topics. So different topics I guess.’ *Girl with inflammatory bowel disease*,* 16 years old.*
		**Q13** ‘I didn’t really have the idea that it was very different, just a bit more detailed, it really went a bit deeper into my thoughts and so on, for example, what really goes on with me… with myself outside the hospital, so to speak.’ *Girl with juvenile rheumatism*,* 18 years old.*
		**Q14** ‘It took a little longer, the conversation. And it wasn’t just about my asthma and whether I was taking my puffs [inhalation medication] properly and so on. But also about the rest.’ *Girl with asthma*,* 10 years old.*
	**Content**	**Q15** ‘Yeah, I liked it. There are questions in there, not all not actually related to your diabetes, so I liked it.’ *Boy with diabetes*,* 15 years old.*
	**Result(s)**	**Q16** ‘Because that was also a bit of a relief for me that I can just, well, mention it again: this is actually something that bothers me. So yeah.’ *Girl with benign tumor*,* 18 years old.*
		**Q17 ‘**But the spider web chart is just… like you can take this with you to, for example, a doctor whom you have spoken to less often or speak to less often, for example my pulmonologist, whom I see much less often: I see him five times a year. Taking this with me would be useful to really feel that I… that I am understood. Because I sometimes miss that with them.’ B*oy with asthma*,* 15 years old.*
		**Q18** ‘Because, for example, after certain things, [name of HCP] also said: well, maybe it would be useful to involve a psychologist. So that is actually very handy that in that way some things do come out that I can get help with, so to speak.’ *Girl with juvenile rheumatism*,* 18 years old.*

##### Awareness of health as a broad concept

Children, especially teenagers, mentioned reflection on their health while using the dialogue tool, contemplating why certain domains had high or low scores (Q7-8). They believed repeated use of the tool could offer deeper insights over time. Most children indicated the frequency of tool use should depend on life circumstances, like changes in their chronic condition or significant events such as starting a new school year. To be able to effectively monitor progress on their health goals, children suggested that there should be sufficient time in between consultations in which the dialogue tool was used. Another topic children spontaneously discussed regarding the dialogue tool was their expectations about the questionnaire outcomes, particularly the overall scores on the spider web chart. Some children shared the feelings they experienced outcomes matched or did not match their expectations (Q9-10). Most children identified with the outcomes displayed on the spider web chart, and a few had specifically discussed this with their HCP.

##### Person-centered consultation

Most children had positive experiences using the MPH dialogue tool, and enjoyed the conversation because it was something new or because it addressed topics beyond their medical condition. However, some faced difficulties when explaining scores in certain domains (Q11). Few children did not experience a difference in the consultation, and one child found it “boring”. In contrast, most children did experience a change in their consultation. They recounted deeper and more personal discussions with their HCPs about various aspects of their lives beyond their chronic condition including their thoughts, feelings, or the condition’s impact (Q12-13). One child, who had a positive experience, mentioned that the conversation took longer (Q14).

Children often discussed their spider web chart results and specific dimensions with their parents or caregivers, particularly *‘My Body*,’ *‘My Feelings & Thoughts*,’ and *‘Now & Later*.’ They evaluated their scores with HCPs by discussing strengths and areas needing improvement.

Many children revealed that they discussed topics they would not typically bring up with their HCPs, and a significant majority felt they were able to discuss what mattered most to them. Some received helpful guidance, (medical) advice or assistance from their HCP in finding solutions. One child felt relieved to open up about her concerns, and about a quarter of all children believed their HCPs now had a better understanding of their priorities and personal challenges (Q16-18).

## Discussion

Our study provides a detailed analysis of the barriers and facilitators associated with the implementation of the MPH dialogue tool in pediatric chronic care, highlighting the person-centered benefits of its use. We gathered quantitative (MIDI questionnaire) and qualitative data from HCPs and obtained qualitative insights from children focusing on the tool’s effectiveness in promoting directorship, awareness of health as a broad concept, and person-centeredness of consultations.

HCPs expressed six facilitators within the user and intervention domain, indicating the tool’s relevance, user-friendliness, and potential to promote a broader view on health among children. Furthermore, they considered the tool relevant to develop a deeper understanding of their patients and facilitate richer conversations. The experiences reported by HCPs in our study align with those found in the implementation of narrative tools, that have shown to enhance communication, promote empathy, and deepen patient understanding [24, 25]. However, they also recognized seven barriers in the organization and innovation domains. HCPs expressed a need for more structured guidance to navigate conversations effectively, and underscored that training significantly influenced the ability to utilize the tool successfully. They articulated concerns regarding the tool’s integration into their existing workflow. Organizational hurdles were highlighted, including time allocation and the availability of material resources. To successfully deploy this innovation, there is a need for institutional support in bridging these gaps.

Children expressed their ability to influence consultation agendas and choose discussion topics using the MPH tool. The tool facilitated goal setting and pinpointing of areas for health improvement, highlighting its role in promoting directorship. Through the MPH dialogue tool, they engaged in introspective reflections about their health, enhancing awareness of their health status in the broader sense. It emerged not just as an informational instrument but as a catalyst for more personalized and in-depth dialogues. The tool enabled conversations that go beyond the scope of a medical condition towards a more comprehensive health discourse, while focusing on the children’s own priorities and challenges.

Our findings align with the broader consensus in pediatrics underscoring the value of person-centered tools for a comprehensive approach to health, intertwining physical, psychological, and social development [26, 27]. Tools can be instrumental in augmenting children’s active engagement and communication, a cornerstone for effective shared decision-making [11, 28–30]. The MPH dialogue tool focuses on personalized health narratives and needs, underlining the relevance of developing and embracing a listening-oriented role to enhance children’s engagement in health conversations [31]. To support this shift, HCPs should be provided with training in navigating this “alternative dialogue” [19]. The MPH dialogue tool’s focus on personalized health narratives aligns with Narrative-Based Medicine (NBM), which emphasizes understanding patients’ stories as a key tool for needs assessment [32]. Both the MPH dialogue tool and NBM foster deeper patient engagement and recognize that communication can occur through non-verbal channels, such as drawings or pictures [32]. The MPH tool for children, for instance, uses a spider web visual chart to help them express their health experiences more effectively. However, it is important to recognize that the spider web chart’s visual appeal can inadvertently put a focus on numerical values – a concern mainly noted by HCPs. While this quantification can be insightful for some, it can be confrontational for others. Therefore, investigating different visualization methods to diminish the focus on numerical scores could be advantageous.

HCPs are generally confident in using the MPH dialogue tool, but reported obstacles related with time constraints and integration in the (predetermined) consultation agenda. In addition to training on how to integrate and use the dialogue tool, HCPs may benefit from more organizational support. This could be realized through targeted logistical assistance or processes expressly devised for the tool’s integration. Introducing ambassadors could promote ongoing application of the MPH dialogue tool and foster collective learning. They could, for instance, be responsible for advocating the tool’s use, providing guidance and support as needed, addressing challenges during implementation, sharing best practices, and gathering feedback to improve the process. In the realm of healthcare innovation, such supports are vital. The tool’s integration with electronic patient records enhances accessibility and consultation efficiency, facilitating long-term health trend tracking. This is especially important as HCPs have reported that the tool is particularly valuable in consultations with children with a chronic condition.

After addressing the identified barriers, longitudinal studies will be crucial to assess the long-term impact of the MPH dialogue tool on pediatric care quality, including its cost-efficiency and effect on healthcare demand. Additionally, the tool’s influence on children with chronic conditions throughout their healthcare journey should be evaluated, with a particular emphasis on boosting patient autonomy and empowerment. It is crucial to determine the specific decision-making moments at which the utilization of the tool proves most beneficial. Lastly, further analysis of parents’ role in consultations using the MPH dialogue tool is vital, particularly considering their significant influence on their child’s health beyond the episodic healthcare visit. Including parents more effectively could further enhance the focus on person-centered goals.

This study combined quantitative and qualitative measures to provide a comprehensive understanding of the child-version of the MPH dialogue tool’s implementation and effectiveness in pediatric clinical practice. Nevertheless, limitations were present, including a relatively small sample size, the inclusion of only Dutch patients, and the potential for selection bias due to voluntary participation of highly motivated HCPs and patients. This may also limit the generalizability of the findings. The predominance of female HCPs in the sample could also have influenced the results. Recall bias is a concern, considering the timing of HCP interviews post-implementation and prior to the questionnaire. Additionally, responses from HCPs and children might be influenced by social desirability bias. Finally, as this study focused on short-term outcomes, further research is necessary to evaluate the long-term impact of implementation of the MPH tool for children. It is important to acknowledge that the MPH dialogue tool is not yet available in all languages and is not appropriate for use with children who have cognitive or sensory disabilities. Future research should prioritize including these populations to enhance its accessibility and effectiveness.

## Conclusion

This study’s mixed-methods approach provided comprehensive insights into the implementation and effectiveness of the My Positive Health (MPH) dialogue tool in pediatric care. Results of the study reveal facilitators like its relevance and user-friendliness and barriers such as organizational constraints. The tool proved beneficial in fostering person-centered care, particularly for children with chronic conditions, by promoting active participation and health awareness. Children felt empowered to guide discussions and engage in self-reflection, enhancing the depth of healthcare dialogues, while HCPs recognized benefits in deepening patient relationships. However, HCPs also faced challenges in integrating the tool into existing practice due to time constraints and consultation structures. To effectively integrate the MPH dialogue tool into routine clinical use and fully realize its potential in enhancing pediatric patient engagement and healthcare quality, it is crucial to address implementation barriers, provide organizational support, and evaluate its impact on the longer term.

## Supplementary Information

Supplementary Material 1. Interview guides.

Supplementary Material 2. Overview of the coding process.

Supplementary Material 3. Determinants for implementing the MPH dialogue tool (adapted MIDI-questionnaire).

Supplementary Material 4. StaRI checklist.

Supplementary Material 5. SRQR checklist.

## Data Availability

The datasets used and/or analyzed during the current study are available from the corresponding author on reasonable request.
