# Supplementary material for: Implementing the My Positive Health dialogue tool for children with a chronic condition: barriers and facilitators
Source: BMC Pediatr. 2025 Mar 5;25:168. doi: 10.1186/s12887-024-05258-0 (PMC11881396; doi:10.1186/s12887-024-05258-0)
Supplement: Supplementary file 1 — Supplementary Material 1. Interview guides. [file 12887_2024_5258_MOESM1_ESM.docx]

**SUPPLEMENT A - Interview guides [Originally in Dutch, translation provided in English]**

1. Interview guide health care providers

**Introductie**- Voorstellen onderzoeker(s)

- Toestemming (video/geluidsopname)

- Duur interview

- Verwerken gegevens anoniem

- Vraag onduidelijk? Geef het aan!

**Persoonsgegevens**- Functie en aandachtgebied?

**Open vraag:**- *Hoe vond u het om de Kindtool/het spinnenweb tijdens uw spreekuur te gebruiken?*- *Welke kinderen selecteerde u om te benaderen voor deelname aan het (implementatie) onderzoek?*

**Topics/determinanten m.b.t. de innovatie**

- Compleetheid (MIDI determinant 3)

- Complexiteit (MIDI determinant 4)
- Congruentie huidige werkwijze (MIDI determinant 5)
- Relevantie cliënt (MIDI determinant 7)

**Topics/determinanten m.b.t. de gebruiker**

- Persoonlijk voordeel/nadeel (MIDI determinant 8)
- Uitkomstverwachting: belangrijkheid en waarschijnlijkheid doelstelling (MIDI determinant 9)
- Taakopvatting (MIDI determinant 10)
- Sociale steun (MIDI determinant 13)
- Eigen effectiviteitsverwachting (MIDI determinant 16)

**Training:** kennis, vaardigheden, expertise
- Kennis (MIDI determinant 17)
- Informatieverwerking (MIDI determinant 18)

**Topics/determinanten m.b.t. de omgeving**

- Steun management (MIDI-determinant 19)
- Tijd (MIDI determinant 23)
- Beschikbaarheid van informatie (over gebruik innovatie) (MIDI determinant 27)

**Open vraag:***Wat kan er verbeterd worden (aan de implementatie/ het implementatieproces/ de innovatie)?*

**Afsluiting**
- Bedankt
- Vragen/opmerkingen?
- Contactgegevens arts-onderzoeker
- Wat gebeurt er met uw gegevens?

**Introduction**

- Introduction of researcher(s)

- Consent for video/audio recording

- Interview duration

- Anonymous data processing

- If a question is unclear, please say so!

**Personal information**

- Role and subspecialty?

**Open question:**

- *How was it for you to use the MPH dialogue tool/ spiderweb chart during your consultations?*

- *Which children did you select to approach for participation in the (implementation) study?*

**Topics/determinants related to the innovation**

- Completeness (MIDI determinant 3)

- Complexity (MIDI determinant 4)

- Congruence current practice (MIDI determinant 5)

- Client relevance (MIDI determinant 7)

**Topics/Determinants Related to the User**

- Personal benefit/drawback (MIDI determinant 8)

- Outcome expectations: importance and likelihood of objectives (MIDI determinant 9)

- Task perception (MIDI determinant 10)

- Social support (MIDI determinant 13)

- Self-efficacy expectation (MIDI determinant 16)

**Training**: Knowledge, Skills, Expertise

- Knowledge (MIDI determinant 17)

- Information Processing (MIDI determinant 18)

**Topics/determinants related to the environment**

- Management Support (MIDI determinant 19)

- Time (MIDI determinant 23)

- Availability of Information (about use of innovation) (MIDI determinant 27)

**Open Question:**

*What improvements can be made (to the implementation (process)/ innovation)?*

**Closing**

- Thanks

- Any questions/comments?

- Contact details of the physician-researcher

- What will happen with your data?

2. Interview guide for children

*Groen = vragen voor kinderen van 8 -12 jaar*

*Blauw = vragen gericht op effectiviteit*

**Introductie**

- Voorstellen
- Informed consent
- Toestemming (geluid/video)opname?
- Duur interview
- Verwerken gegevens anoniem
- Vraag onduidelijk? Geef het aan!

**Persoonsgegevens**

- Leeftijd
- Ga je naar school? *Zo ja: welke groep (basisonderwijs) of welke klas en niveau (middelbaar onderwijs)?*
- Voor welke ziekte(n)/aandoening(en) kom je in het ziekenhuis? *Hoe lang kom je al in het ziekenhuis?* *Hoe vaak kom je naar het ziekenhuis?*

**Vragen**

- Als jij naar de dokter gaat, hoe gaat dat? Vertel eens.
- Wat gebeurt er als je bij de dokter bent? of: Hoe gaat normaal het gesprek met de dokter?
  - *Hoe was dat nu?*
- Wat wilde de dokter van jou weten?
- Hoe was het voor jou om de Kindtool (en het (spinnen)web) te gebruiken tijdens het gesprek met de dokter?
  - *Herkende je jezelf in de uitkomst/in het spinnenweb?*
  - *Horen alle aspecten (wat jou betreft) bij gezondheid?*
  - *Is de Kindtool een fijne manier om dit gesprek aan te gaan?*
- Denk je wel eens na over je gezondheid?
- Hoe denk je nu over je gezondheid (na Kindtool)
- Had je van tevoren bedacht wat je wilde bespreken met de dokter?
- Heb je thuis iets gedaan voordat je naar de dokter ging?
  - *Praat je er van tevoren over (met iemand)?*
  - *Heeft de Kindtool daarbij geholpen?*
  - *Is wat je met de dokter wilde bespreken veranderd door dat je de kindtool hebt ingevuld? Kan je daar wat meer over vertellen?*

*Green = questions for children aged 8-12 years*

*Blue = questions focused on effectiveness*

**Introduction**

- Introductions
- Informed consent
- Permission for audio/video recording?
- Duration of interview
- Anonymous data processing
- If a question is unclear, please say so!

**Personal information**

- Age
- Do you go to school? *If yes, which grade (primary school), or which class and level (secondary school)?*
- What illness(es)/condition(s) do you visit the hospital for? *For how long have you been coming to the hospital? How often do you visit the hospital?*

**Questions**

- When you go to the doctor, how does it go? Tell me about it.
- What happens when you are with the doctor? Or: How does a normal conversation go?
  - *How was it this time?*
- What did the doctor want to know from you?
- How was it for you to use the MPH tool (and the spiderweb chart) during the conversation with the doctor?
  - *Did you recognize yourself in the outcome/ spider web chart?*
  - *Do all aspects (in your opinion) relate to health?*
  - *Is the MPH tool a nice way to have this conversation?*
- Do you ever think about your health?
- How do you view your health now (after MPH tool for children)?
- Had you thought beforehand about what you wanted to discuss with the doctor?
- Did you do anything at home before going to the doctor?
  - *Do you talk about it to someone beforehand?*
  - *Did the MPH tool help with that?*
  - *Has what you wanted to discuss with the doctor changed because you filled in the MPH tool? Can you tell me more about that?*
- Voelde je je gehoord bij de dokter of verpleegkundige? *Evt. had je het gevoel dat hij/zij naar jou luisterde en jou begreep?*
  - *Waar merkte je dat aan?*
- Heb jij kunnen vertellen/bespreken wat voor jou belangrijk is?
- Wat zou je ervan vinden als we je zouden vragen de Kindtool voor een volgende afspraak opnieuw in te vullen?
- Zou je andere kinderen aanraden om het te doen?
  - *Waarom?*

**Innovatie, tijd en overig**

- Wat vond je van de informatie vooraf?
  - *Was de informatie vooraf begrijpelijk? Snapte je goed wat je moest doen?*
- Sommige kinderen vinden het best moeilijk om de vragenlijst in te vullen, andere kinderen vinden het juist makkelijk. Hoe was dat voor jou?
  - *Heb je de vragenlijst alleen ingevuld of heb je hulp gevraagd? Zo ja, aan wie?*
- Hoeveel tijd heeft het invullen van de vragenlijst jou gekost?
  - *Vond je dat het invullen van de vragenlijst (Kindtool) veel (extra) tijd kostte?*
- Wat kan er (nog) verbeterd worden?

**Afsluiting**

- Bedankt
- Nog vragen/opmerkingen?
- Vragenlijsten online (welk emailadres)?
- Deelname focusgroep?
- Contactgegevens arts-onderzoeker
- Wat gebeurt er met jouw gegevens?
- Did you feel heard by the doctor or nurse? *If necessary:* *did you feel that he/she listened to you and understood you?*
  - *How could you tell?*
- Were you able to talk/discuss what is important to you?
- How would you feel if we asked you to fill out the MPH tool for children again for the next appointment?
- Would you recommend other children to do/ use it?
  - *Why?*

**Innovation, Time, and Others**

- What did you think of the information?
  - *Was the information understandable? Did you understand what you had to do?*
- Some children find it quite difficult to fill in the questionnaire, while others find it easy. How was it for you?
  - *Did you fill in the questionnaire alone, or did you ask for help? If so, from whom?*
- How much time did it take you to fill in the questionnaire?
  - *Did you find that filling in the questionnaire (MPH tool) took a lot of (extra) time?*
- What can be improved?

**Closing**

- Thank you
- Any questions/comments?
- Questionnaires online (which email address)?
- Participation in focus group?
- Contact details of the physician-researcher
- What will happen with your data?
