# Supplementary material for: Implementing the My Positive Health dialogue tool for children with a chronic condition: barriers and facilitators
Source: BMC Pediatr. 2025 Mar 5;25:168. doi: 10.1186/s12887-024-05258-0 (PMC11881396; doi:10.1186/s12887-024-05258-0)
Supplement: Supplementary file 2 — Supplementary Material 2. Overview of the coding process. [file 12887_2024_5258_MOESM2_ESM.docx]

**SUPPLEMENT B – Overview of the coding process**

The final code tree comprised of 25 codes and 37 subcodes. The interview transcripts were coded in batches of 3-5 interviews, with batches 1-5 coded during the first implementation cycle and batches 6-8 during the second. The majority of codes (94%) were introduced in the first cycle, as illustrated in Figure 1. Code saturation appeared to be reached after the fifth batch. Nonetheless, in the second cycle, one additional code and three new subcodes emerged.

**Figure 1. Timing of code development**

During analysis of the interviews of the first implementation cycle, 8 code definitions were altered during the coding process, 6 after analysis of the first batch of interviews and two more after the third batch. Five (sub)codes were eventually merged into either existing codes or novel codes due to overlapping definitions. During the second implementation cycle no code definitions were altered, no (sub)codes were merged and no code descriptions were changed.
