# Supplementary material for: Implementing the My Positive Health dialogue tool for children with a chronic condition: barriers and facilitators
Source: BMC Pediatr. 2025 Mar 5;25:168. doi: 10.1186/s12887-024-05258-0 (PMC11881396; doi:10.1186/s12887-024-05258-0)
Supplement: Supplementary file 3 — Supplementary Material 3. Determinants for implementing the MPH dialogue tool (adapted MIDI-questionnaire). [file 12887_2024_5258_MOESM3_ESM.docx]

**SUPPLEMENT C – Determinants for implementing the MPH dialogue tool (adapted MIDI-questionnaire)**

| Determinants for implementing the MPH dialogue tool as measured by the Measurement Instrument for Determinants of Innovations (*n*=18) | | | | | | | | |
| --- | --- | --- | --- | --- | --- | --- | --- | --- |
|  | MIDI scale and items |  |  |  | **Disagree/totally disagree** | **Neutral** | | **Agree/totally agree** |
|  |  | | |  | (%) | (%) | | (%) |
|  | **Innovation/intervention** |  |  |  |  |  | |  |
| 1 | Procedural clarity: the MPH dialogue tool clearly describes the activities I should perform and in which order | | |  | 16,7 | 16,7 | | 66,7 |
| 2 | Correctness: the MPH dialogue tool is based on information that is reliable | | |  | 11,1 | 16,7 | | 72,2 |
| 3 | Completeness: the MPH dialogue tool provides all the information and materials needed to work with it properly | | |  | **27,8** | 11,1 | | 61,1 |
| 4 | Complexity: the MPH dialogue tool is too complex for me to use |  |  |  | **100** | 0 | | 0 |
| 5 | Compatibility: The MPH dialogue tool fits well with how I am used to working. | | |  | **22,2** | 16,7 | | 61,2 |
| 6 | Observability: the outcomes of using the MPH dialogue tool are clearly observable | | |  | 16,7 | 33,3 | | 50 |
| 7 | Relevance for patient: I think the MPH dialogue tool is relevant for my patients | | |  | 0 | 16,7 | | **83,3** |
|  | **User** |  |  |  |  |  | |  |
| 8a* | Personal benefits: using the MPH dialogue tool helps me better understand what is important to my patients regarding their health | | | | 5,6 | 5,6 | | **88,9** |
| 8b* | Personal benefits: using the MPH dialogue tool helps me develop a broader view of health | | | | 11,1 | 16,7 | | 72,2 |
| 8c* | Personal drawbacks: using the MPH dialogue tool costs me (too) much extra time during consultation hours. | | | | 16,7 | 44,4 | | 38,9 |
| 8d* | Personal drawbacks: by using the MPH dialogue tool, my patients ask me for help in areas where I have insufficient knowledge | | | | 50 | 33,3 | | 16,7 |
| 9a* | Outcome expectations: the patient is (well) prepared for the conversation with the HCP | | | | 0 | 55,6 | | 44,4 |
| 9b* | Outcome expectations: the patient develops a broader view of health | | | | 0 | 5,6 | | **94,4** |
| 9c* | Outcome expectations: the patient is able to raise issues important to his/her health | | | | 5,6 | 22,2 | | 72,3 |
| 9d* | Outcome expectations: the patient develops more control over his/her care process | | | | 0 | 50 | | 50 |
| 10 | Professional obligation: I feel it is my responsibility as a professional to use the MPH dialogue tool | | | | 16,7 | 5,6 | | 77,8 |
| 11 | Patient satisfaction: my patients will generally be satisfied if I use the MPH dialogue tool | | | | 0 | 22,2 | | 77,8 |
| 12 | Patient cooperation: patients will generally cooperate if I use this innovation | | | | 0 | 22,2 | | 77,8 |
| 13 | Social support: I can count on adequate assistance from my colleagues if I need it to use the MPH dialogue tool | | | | 5,6 | 11,1 | | **83,4** |
| *14* | *Descriptive norm: colleagues' observed behavior; degree to which colleagues use the innovation* | | | | - | - | | - |
| *15* | *Subjective norm: the influence of important others on the use of the innovation* | | | | - | - | | - |
| 16 | Self-efficacy: do you think you would be able to apply the MPH dialogue tool during your consultation hours? ^1^ | | | | 0 | 33,3 | | 66,7 |
| 17a | Knowledge: I have sufficient knowledge to be able to use the MPH dialogue tool | | | | 0 | 16,7 | | **83,4** |
| 18 | Awareness of content: to what extent are you informed about the content of the MPH dialogue tool^2^ | | | | **I've glanced through it (%)**  27,8 | | **I have read it thoroughly (%)**  72,2 | |
|  |  | | | |  |  |  |  |
|  |  | | | |  |  |  |  |
|  | **Organization** |  |  |  | **No (%)**  0 | **I don’t know (%)**  77,8 | | **Yes (%)**  22,2 |
| 19 | Formal ratification by management: Is the use of the MPH dialogue tool supported by management?^3^ | | | |  |  |  |  |
|  |  | | | |  |  |  |  |
|  |  | | | | **Disagree/totally disagree (%)** | **Neutral (%)** | | **Agree/totally agree (%)** |
| *20* | *Replacement when staff leave* | | | | - | - | | - |
| 21 | There are enough people in our organization to use the innovation as intended | | | | 5,6 | 33,3 | | 61,2 |
| 22 | Financial resources: there are enough financial resources available to use the MPH dialogue tool as intended | | | | 5,6 | 61,1 | | 33,4 |
| 23 | Time available: the time available is sufficient to integrate the MPH dialogue tool into my daily work as intended | | | | **33,4** | 33,3 | | 33,3 |
| 24 | Material resources and facilities: our organization provides me with enough materials and other resources or facilities | | | | **22,3** | 44,4 | | 33,3 |
| 25 | Coordinator: in my organization, one or more people have been designated to coordinate the process of implementiation^4^ | | | | 27,8 (no) |  | | 72.2 (yes) |
| 26 | Unsettled organization: are there any other changes in the organization affecting the implementation now or in the foreseeable future?^4^ | | | | 44,4 (no) |  | | 55,6 (yes) |
| 27 | Information accessible: I have easy access in my organization to information on the use of the MPH dialogue tool for children | | | | **22,3** | 27,8 | | 50 |
| 28 | Performance feedback: In my organization, there is regular feedback on the progress of the implementation of the MPH dialogue tool | | | | **61,1** | 22,2 | | 16,7 |
|  | **Socio-political context** |  |  |  |  |  | |  |
| *29* | *Legislation and regulations* |  |  |  | - | - | | - |

HCP: health care professional; MPH: My Positive Health
Numbers in **bold** represent a HCP-reported barrier (≥ 20% disagrees/totally disagree) or facilitator (≥ 80% agrees/totally agrees). Determinants in *italics* were omitted from the questionnaire.
^1^: Response scale: (1) most definitely not (2) definitely not (3) perhaps not, perhaps (4) definitely (5) most definitely
^2^: Response scale: (1) I'm not familiar with the innovation (2) I'm familiar with the innovation, but I haven't read it through (yet) (3) I'm familiar with the innovation and I've glanced through it (4) I'm familiar with the innovation and I have read through it thoroughly.
^3^: Response scale: (1) no (2) yes (3) I don’t know
^4^: Response scale: (1) no (2) yes
^*^ Facilitators 8 and 9 are items that can be split into multiple questions within the questionnaire. For item 8 (personal benefits/drawbacks) we added several questions with each question focusing on a single personal benefit or drawback. Results show that one of these specific benefits (8a: ‘’using the MPH dialogue tool for children helps me better understand what is important to my patients regarding their health’’) was identified as a facilitator. For item 9, we added questions referring to different potential expected outcomes and one of these expected outcomes (9b “the patient develops a broader view of health”) was identified a facilitator.
